# The long-term survival of patients with III-IVb stage nasopharyngeal carcinoma treated with IMRT with or without Nimotuzumab: a propensity score-matched analysis

**DOI:** 10.1186/s12885-019-6156-5

**Published:** 2019-11-19

**Authors:** Wang Zhi-Qiang, Mei Qi, Li Ji-Bin, You Rui, Liu You-Ping, Sun Rui, Hu Guang-Yuan, Chen Ming-Yuan, Hua Yi-Jun

**Affiliations:** 1 State Key Laboratory of Oncology in South China, Collaborative Innovation Center for Cancer Medicine, Guangzhou, China; 2 Department of Nasopharyngeal Carcinoma, Sun Yat-sen University Cancer Center, 651 Dongfeng Road East, Guangzhou, 510060 China; 3 Department of Oncology, Tongji Hospital, Tongji Medical College of Huazhong University of Science and Techology, Wuhan, China; 4 Department of Clinical Research, Sun Yat-sen University Cancer Center, Guangzhou, China

**Keywords:** Nasopharyngeal carcinoma, IMRT, Chemotherapy, Nimotuzumab, Prognosis

## Abstract

**Background:**

To assess the efficacy of Nimotuzumab in combination with first-line chemoradiotherapy treatment in Chinese patients with primary III-IVb stage nasopharyngeal carcinoma.

**Methods:**

Patients with primary locoregionally advanced nasopharyngeal carcinoma who were treated with intensity-modulated radiotherapy (IMRT) and concurrent cisplatin-based chemotherapy between January 2008 and December 2013 at a single institution were retrospectively reviewed. Group A received at least 6 doses of Nimotuzumab, while Group B did not receive Nimotuzumab. A propensity score matching method was used to match patients from each group in a 1:3 ratio.

**Results:**

In total, 730 eligible patients were propensity matched, with 184 patients in Group A and 546 patients in Group B. Significant differences were not observed in the patient and tumor characteristics between Group A and Group B. At a median follow-up of 74.78 months (range 3.53–117.83 months), locoregional recurrence, distant failure and death were observed in 10.68, 11.10 and 16.03% of all patients, respectively. The estimated 5-year locoregional relapse–free survival, distant metastasis–free survival, progression-free survival and overall survival in the Group A versus Group B were 85.34% versus 89.79% (*P* = 0.156), 93.09% versus 85.61% (*P* = 0.012), 79.96% versus 77.99% (*P* = 0.117) and 88.91% versus 78.30% (*P* = 0.006), respectively.

**Conclusions:**

This nimotuzumab-containing regimen resulted in improved long-term survival of III-IVb stage NPC patients and warrants further prospective evaluation.

## Background

Nasopharyngeal carcinoma (NPC) is a cancer arising from the nasopharynx epithelium. Most new cases occur in Southeast Asia, and it is also endemic in southern China [1–3]. Due to the large population and high morbidity of nasopharyngeal carcinoma (NPC) in South China [4], the number of NPC patients is considerable, and nearly 5000 NPC patients are diagnosed at Sun Yat-sen University Cancer Centre each year. NPC is distinguished from other types of head and neck cancers by its unique sensitivity to both radiotherapy and chemotherapy. The current management of loco-regionally advanced NPC is radiotherapy combined with cisplatin-based concurrent chemotherapy. With the development of modern radiation therapy techniques in recent decades, the treatment outcomes have improved considerably [5]. However, NPC treatment has entered a plateau period, and new strategies or methods are required to achieve further improvements.

EGFR is overexpressed in approximately 90% of squamous cell carcinomas of the head and neck [6–8], and more than 80% of NPC patients overexpress EGFR; moreover, its expression is associated with unfavorable T stage and overall survival [9, 10]. With the development of molecular-targeted therapy, EGFR represents a promising therapeutic target in oncology because of its correlation with aggressive phenotypes, treatment resistance and poor prognosis. Nimotuzumab is a humanized anti-EGFR monoclonal antibody that binds to the extracellular domain of EGFR and inhibits EGF binding, and it is designed to reduce immunoreactivity and enhance radio sensitivity [11]. Nimotuzumab has demonstrated a unique clinical safety profile [12], where anti-tumor activity was observed without severe skin, renal, and gastrointestinal mucosa toxicities commonly associated with EGFR-targeting antibodies [13]. Previous clinical studies of nimotuzumab concurrent with radiotherapy in patients with locally advanced head and neck squamous cell carcinoma reported that the combination was well tolerated and may enhance the radio curability of unresectable head and neck neoplasms [14]. In addition, the side effects from introducing Nimotuzumab to chemoradiotherapy were mild, and this antibody did not affect the normal execution of radiotherapy [15].

In this study, we aimed to assess the efficacy of nimotuzumab combined with radiotherapy in patients with advanced nasopharyngeal carcinoma. The primary endpoint was the evaluation of overall survival and progression-free survival.

## Methods

### Patients

**Fig. 1:**
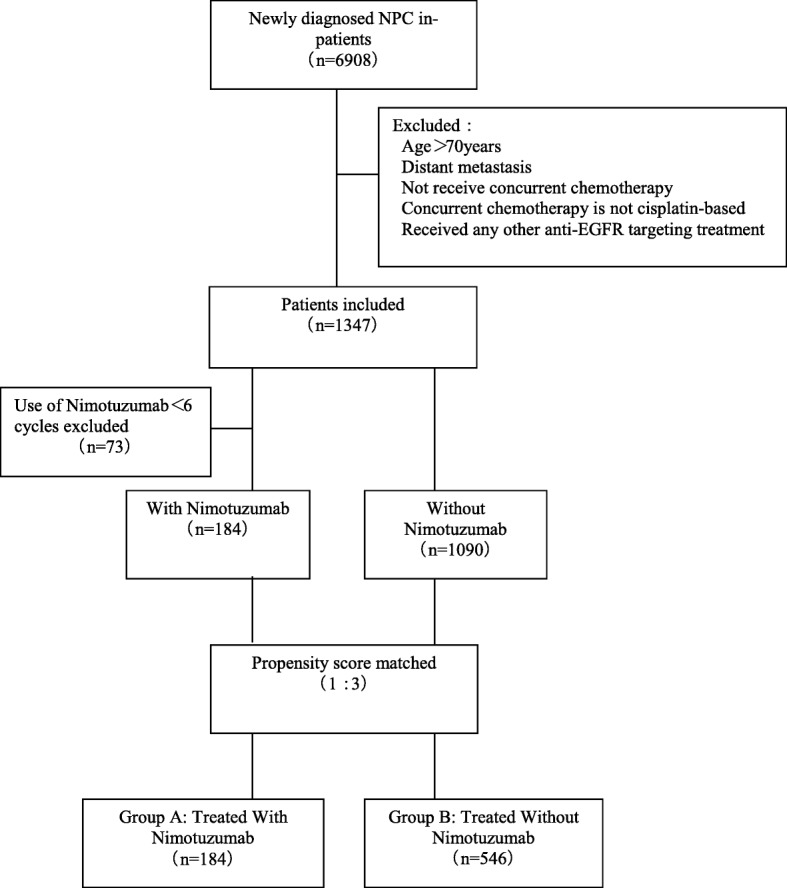
Study flow diagram

The Clinical Research Ethics Committee of Sun Yat-sen University Cancer Center (SYSUCC) approved this retrospective review. We reviewed the inpatient medical records of primary nasopharyngeal carcinoma patients treated with IMRT at SYSUCC between January 2008 and December 2013. A total of 6908 patients were identified, and eligible patients met the following criteria: (i) III-IVb disease stages; (ii) histologically proven nonmetastatic NPC; (iii) Karnofsky Performance Status (KPS) ≥80; (iv) completion of radical radiotherapy; and (v) no previous anti-cancer treatment. The exclusion criteria were as follows: (a) age > 70 years; (b) disease progression during radiotherapy; (c) pregnancy or lactation; (d) lack of concurrent chemotherapy; (e) concurrent chemotherapy is not cisplatin-based; (f) received other anti-EGFR targeting therapy; and (g) previous malignancy or other concomitant malignant disease. The staging workup included an MRI of the head and neck, a chest radiograph, a bone scintigraphy, and an ultrasonography of the abdominal region for all the patients. All the included patients were restaged according to the Seventh Edition of the American Joint Committee on Cancer (AJCC) staging system. From these criteria, 1274 patients were selected for the matched study (Fig. 1).

We performed an analysis of variance as well as a χ^2^ test on the patients’ baseline demographics and clinical characteristics. Variable differences were identified between the two groups, including gender, age, tumor stage (T stage) and node stage (N stage), clinical stage and chemotherapy regime, all of which were identified as prognostic factors for survival outcomes in a previous study. Using propensity scores to adjust for these 6 factors, we created a well-balanced cohort by matching each patient who underwent nimotuzumab treatment with no more than three patients who underwent chemoradiotherapy without nimotuzumab (Table 1). From this stratification process, we selected a total of 730 patients, including 184 patients in the nimotuzumab arm and 546 patients in the no nimotuzumab arm (Table 1). We first conducted case-matched comparisons between the two arms in terms of efficacy and safety in this well-balanced cohort of 730. Subsequently, we conducted univariable and multivariate analyses of the 730 patients.

**Table 1:** Baseline characteristics of patients with NPC treated with or without Nimotuzumab

Characteristic	Nimotuzumab Arm *N* = 184(%)	No Nimotuzumab Arm *N* = 546(%)	*P* value
Gender			0.704
Female	37(20.11)	117(21.43)	
Male	147(79.89)	429(78.57)	
Age, Mean (SD)	43.92 (10.53)	44.1 2(10.62)	0.822
<44	95(51.63)	253(46.34)	
≥ 44	89(48.37)	293(53.66)	
WHO pathology			0.436
I	3(1.6)	11(2.0)	
II	8(4.3)	14(2.6)	
III	173(94.1)	521(95.4)	
T classification			0.966
T1	5(2.72)	17(3.11)	
T2	16(8.70)	42(7.69)	
T3	107(58.16)	317(58.06)	
T4	56(30.42)	170(31.14)	
N classification			0.972
No	19(10.33)	57(10.44)	
N1	75(40.76)	230(42.13)	
N2	73(39.67)	214(39.19)	
N3	17(9.24)	45(8.24)	
Clinical stage			0.937
III	116(63.04)	346(63.37)	
IVa	51(27.72)	155(28.39)	
IVb	17(9.24)	45(8.24)	
Chemotherapy			0.684
Concurrent	61(33.15)	190(34.80)	
Induction + concurrent	123(66.85)	356(65.20)	

### Treatment

#### Radiation therapy

All patients received IMRT. The primary nasopharyngeal gross tumor volume (GTVnx) and the involved cervical lymph nodes were determined based on MRI/CT and/or PET-CT imaging, clinical, and endoscopic findings. The enlarged retropharyngeal nodes together with primary gross tumor volume (GTV) were outlined as the GTVnx on the IMRT plans. The clinical tumor volume (CTV) represents the primary tumor with potential subclinical disease. The first clinical tumor volume (CTV1) was defined as the GTV plus a 0.5–1.0 cm margin (0.2 to 0.3 margin posteriorly) to encompass the high-risk sites of microscopic extension and the whole nasopharynx. Clinical target volume 2 (CTV2) was defined as the CTV1 plus a 0.5–1.0 cm margin (0.2 to 0.3 margin posteriorly) to encompass the low-risk sites of microscopic extension, the level of the lymph node, and the elective neck area (bilateral levels IIa, IIb, III, and Va are routinely covered for all N0 patients, whereas ipsilateral levels IV, Vb, or supraclavicular fossae are also included for N1–3 patients). The prescribed doses were 66–70 Gy to the planning target volume (PTV) of the primary gross tumor volume (GTVnx), 60 Gy to PTV1 (PTV of CTV1), 54 Gy to PTV2 (PTV of CTV2), and 60–66 Gy to PTVnd of the involved cervical lymph nodes in 28 to 33 fractions. All patients were treated once daily, with five fractions administered weekly. The doses to critical structures were within the tolerance range according to the RTOG 0225 protocol, and efforts were made to meet the criteria as closely as possible.

#### Chemotherapy

During the study period, concurrent chemoradiotherapy (CCRT) ± induction chemotherapy (IC) for stage III to IV disease was recommended according to our institutional guidelines. The study-defined concurrent chemoradiotherapy regimen was 80–100 mg/m2 cisplatin on day 1 every 3 weeks for 2–3 cycles or 30 mg/m2 cisplatin weekly. Patients receiving other chemotherapy regimens or who received only one cycle of induction or concurrent chemotherapy were excluded from this study. The study-defined induction chemotherapy regimens included PF (*n* = 161) (80–100 mg/m2 cisplatin on day 1 and 800 mg/m2 /d fluorouracil civ on days 1–5), TP (*n* = 176) (75 mg/m2 docetaxel on day 1 and 75 mg/m2 cisplatin on day 1 or TPF(142) (75 mg/m2 docetaxel on day 1, 75 mg/m2 cisplatin on day 1 and 800 mg/m2 /d fluorouracil civ on days 1–5), and both regimens were repeated every 3 weeks for 2–3 cycles. The reasons for deviating from the institutional guidelines included organ dysfunction suggesting intolerance to chemotherapy, patient refusal, and the discretion of the doctors in individual cases.

#### Nimotuzumab delivery

Nimotuzumab was not recommended for NPC patients by the guideline at that time. Therefore, the use of Nimotuzumab was determined by the patients’ willingness and the doctors’ experience. Intravenous Nimotuzumab was administered at an initial dose of 200 mg weekly during the entire radiation period. A total of 184 patients received full doses of Nimotuzumab.

#### Follow-up

Patient follow-up was measured from the first day of therapy to the last examination or death. Patients were examined at least every 3 months during the first 2 years, with follow-up examinations every 6 months for 3 years or until death. The last follow-up date was 20 April 2019.

The Common Terminology Criteria for Adverse Events (version 4.0) was used to evaluate chemotherapy-related toxic effects, and the Late Radiation Morbidity Scoring Criteria of the Radiation Therapy Oncology Group was used to evaluate radiotherapy-related toxic effects [16]. Acute toxicities were defined as those occurring either during the course of IMRT or within 90 days of its completion.

### Statistical analysis

Distant metastasis–free survival (DMFS) and locoregional relapse–free survival (LRRFS) were calculated from day 1 after completion of treatment to the first distant metastasis and locoregional relapse, respectively. Progression–free survival (PFS) was calculated from day 1 after completion of treatment to locoregional relapse, distant relapse or tumor-related death, whichever occurred first. Overall survival (OS) was calculated from day 1 after completion of treatment to the last examination or death.

The clinic-pathologic characteristics of participants are described, and the differences of these characteristics between the Nimotuzumab group and non-Nimotuzumab group were compared by the *χ*^2^ test for categorical variables and the *t*-test for continuous variables. Logistic regression analysis was used to identify confounders between the treatment groups. A propensity score matching method was used. Propensity scores were calculated based on the identified potential confounders and other important factors, such as tumor stage, and then each patient was assigned a score. Using a caliper width of 0.2, 1:3 matching was performed between patients in the Nimotuzumab group and non-Nimotuzumab group based on the propensity scores.

LRRFS, DMFS, PFS and OS were calculated using the Kaplan-Meier method. The differences in LRRFS, DMFS, PFS and OS between the two groups were tested using the log-rank test. Multivariate analysis was performed using the Cox proportional hazards models. All statistical analyses were performed using SPSS 21.0 statistical software (Chicago, IL, USA). A value of *P* < 0.05 was considered statistically significant.

## Results

### Patient characteristics

Patient characteristics are detailed in Table 1. A total of 6908 consecutive NPC patients who were treated with IMRT between January 2008 and December 2013 at SYSUCC were analyzed, and 1274 patients were eligible for propensity score matching as shown in Fig. 1. Gender, age, T-category, N-category, clinical stage and chemotherapy regime (IC alone and IC + CCRT) were used to generate a propensity score model (Fig. 1).

In total, 730 patients were propensity matched in this study to create two groups: Group A, which received Nimotuzumab, included 184 cases; and Group B, which did not receive Nimotuzumab, included 546 cases. Among the 730 patients, 154 were female and 576 were male. All 730 patients received cisplatin-based concurrent chemotherapy, and 479 received two courses of induction chemotherapy. The characteristics of the patients were well balanced between the propensity-matched groups. The median dose delivered during the initial course of radiation was 70 Gy (range, 66–80 Gy).

The mean age at the time of reirradiation was 43.92 years (SD = 10.53) for Group A and 44.12 years (SD = 10.62) for Group B. At a median follow-up of 74.78 months (range 3.53–117.83 months), the 1, 3, and 5-year follow-up rates were 99.6, 96.7 and 90.5%, respectively.

### Efficacy and safety

At the median follow-up of 74.78 months (range 3.53–117.83 months), 117 deaths (16.03%) had occurred. At the time of the analysis, 68 patients had locoregional failure (9.32%), 10 showed locoregional failure and distant metastases (1.34%), and 71 developed distant metastases (9.73%).

**Fig. 2:**
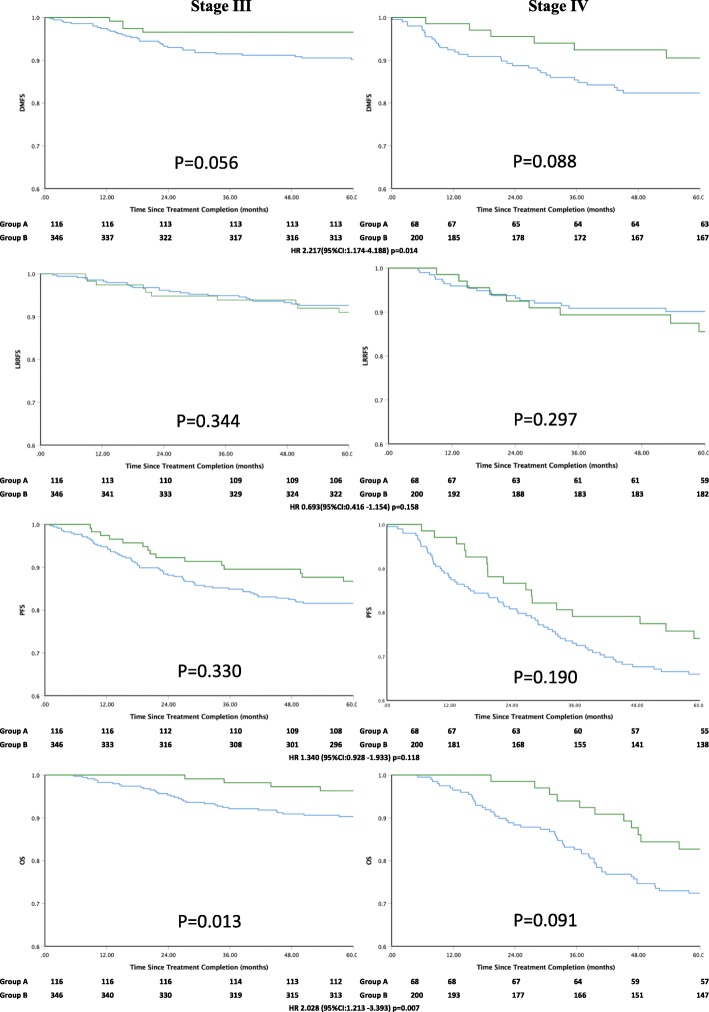
Kaplan-Meier plots of distant metastasis-free survival, locoregional relapse–free survival, progression-free survival, and overall survival of NPC patients treated with (green lines) or without Nimotuzumab (blue lines) according the clinical stage

The 5-year DMFS, LRRFS, PFS and OS rates for Group A vs. Group B were 93.90% vs. 85.61% (*P* = 0.012), 85.34% vs. 89.79% (*P* = 0.156), 79.96% vs. 77.99% (*P* = 0.117) and 96.33% vs. 85.97% (*P* = 0.006), respectively (Table 2). Significant differences in DMFS and OS were observed between Group A and Group B, although differences in LRRFS and PFS were not observed. The 5-year DMFS, LRRFS, PFS and OS according to clinical stage were calculated, and significant differences were only observed in OS for stage III. The survival curves are shown in Fig. 2.

**Table 2:** Five-year survival rates of the 730 NPC patients

	All (*N* = 730)	Nimotuzumab Arm (N = 184)	No Nimotuzumab Arm (N = 546)	Chi-square	*P* value
DMFS	87.49%	93.09%	85.61%	6.343	*0.012*
III	89.81%	94.59%	88.22%		
IV	83.43%	90.56%	80.93%		
LRRFS	88.60%	85.34%	89.79%	2.012	0.156
III	90.16%	87.22%	91.21%		
IV	85.71%	82.00%	87.17%		
PFS	78.47%	79.96%	77.99%	2.459	0.117
III	78.72%	81.27%	77.88%		
IV	63.15%	70.90%	60.52%		
OS	80.96%	88.91%	78.30%	7.565	*0.006*
III	88.53%	96.33%	85.97%		
IV	67.69%	76.24%	64.79%		

*OS* overall survival, *DMFS* distant metastasis–free survival, *LRRFS* locoregional relapse–free survival, and *PFS* progression-free survival

Table 3 displays the acute toxicities of the 730 patients. Significant differences were not observed in the hematological toxicities, and significant differences were not observed between the two groups in terms of hepatoxicity, nephrotoxicity, and gastrointestinal reactions, including nausea, vomiting, and diarrhea.

**Table 3:** Acute toxicities in the 730 NPC patients

Acute Toxicity	Nimotuzumab Arm *N* = 184(%)	No Nimotuzumab Arm *N* = 546	*P* value
Anemia			0.920
G0-G1	137(74.5)	416(76.2)	
G2	36(19.6)	98(17.9)	
G3	9(4.0)	27(4.9)	
G4	2(1.1)	5(0.9)	
Thrombocytopenia			0.541
G0-G1	164(89.1)	464(85.0)	
G2	16(8.7)	58(10.6)	
G3	4(2.2)	21(3.8)	
G4	0(0.0)	3(0.5)	
Leukopenia			0.845
G0-G1	122(66.3)	370(67.8)	
G2	33(17.9)	101(18.5)	
G3	27(14.7)	7113.0()	
G4	2(1.1)	4(0.7)	
Neutropenia			0.154
G0-G1	139(75.5)	386(70.7)	
G2	39(21.2)	118(21.6)	
G3	5(2.7)	38(7.0)	
G4	1(0.5)	4(0.7)	
Skin reaction			0.434
G0-G1	126(68.5)	381(69.8)	
G2	46(25.0)	117(21.4)	
G3	12(6.5)	48(8.8)	
Mucositis			0.728
G0-G1	67(36.4)	189(34.6)	
G2	73(39.7)	228(41.8)	
G3	38(20.7)	102(18.7)	
G4	6(3.3)	27(4.9)	
Nausea			0.394
G0-G1	62(37.8)	193(35.3)	
G2	71(43.3)	214(39.2)	
G3	28(17.1)	126(23.1)	
G4	3(1.8)	13(2.4)	
Vomiting			0.990
G0-G1	138(75.0)	412(75.5)	
G2	24(13.0)	68(12.5)	
G3	20(10.9)	61(11.2)	
G4	2(1.1)	5(0.9)	
Diarrhea			0.495
G0-G1	146(79.3)	437(80.0)	
G2	31(16.8)	97(17.8)	
G3	7(3.8)	12(2.2)	
Hepatotoxicity			0.532
G0-G1	137(74.5)	410(75.1)	
G2	32(17.4)	80(14.7)	
G3	15(8.2)	56(10.3)	
Nephrotoxicity			0.770
G0-G1	167(90.8)	505(92.5)	
G2	12(6.5)	29(5.3)	
G3	5(2.7)	12(2.2)	
Weight loss			0.964
G0-G1	14,478.3()	420(76.9)	
G2	36(19.6)	112(20.5)	
G3	4(2.2)	14(2.6)	

### Prognosis

The overall survival (OS) of the 730 cases were analyzed by univariate and multivariable cox regression models, respectively. We included sex, age, T stage, N stage, clinical stage, nimotuzumab treatment or not and concurrent chemotherapy (with or without induction chemotherapy) in the model. The results showed that the T stage, N stage, clinical stage and nimotuzumab or not factors had prognostic significance for OS (Table 4). The multivariate analysis indicated that N stage and nimotuzumab treatment were independent prognostic factors for DMFS and OS (Table 5). Patients with advanced N stage had a poorer prognosis and those who received nimotuzumab had significantly better 5-year OS rates compared with than those who did not receive nimotuzumab (88.91% versus 78.30%, P<0.01) (Table 2).

**Table 4:** Prognostic factors associated with overall survival based on univariate cox regression models (N = 730)

	DMFS				LRRFS				PFS				OS			
	B	SE	HR(95%CI)	*P*	B	SE	HR(95%CI)	*P*	B	SE	HR(95%CI)	*P*	B	SE	HR(95%CI)	*P*
Gender																
Female		1				1				1				1		
Male	−0.943	0.372	0.389(0.188–0.808)	0.011	0.189	0.280	1.208(0.697–2.092)	0.500	−0.270	0.198	0.764(0.518–1.126)	0.173	−0.570	0.269	0.566(0.3340.959)	0.034
Age																
<44		1				1				1				1		
≥ 44	−0.106	0.223	0.900(0.581–1.393)	0.636	− 0.380	0.248	0.684(0.420–1.111)	0.125	− 0.242	0.152	0.785(0.582–1.058)	0.112	− 0.288	0.187	0.750(0.519–1.083)	0.125
Tumor stage																
T1		1				1				1				1		
T2	0.681	0.486	1.975(0.763–5.116)	0.161	−0.145	0.736	0.865(0.204–3.661)	0.844	0.050	0.396	1.051(0.484–2.285)	0.900	0.050	0.429	1.052 (0.454–2.438)	0.906
T3	−0.213	0.450	0.808(0.335–1.952)	0.636	0.261	0.408	1.299(0.583–2.891)	0.522	−0.126	0.277	0.882(0.513–1.517)	0.650	−0.584	0.358	0.557 (0.276–1.125)	0.103
T4	−0.292	0.244	0.747(0.463–1.205)	0.232	−0.373	0.267	0.689(0.408–1.162)	0.162	−0.577	0.162	0.562(0.409–0.772)	0.000	−1.011	0.200	0.364 (0.246–0.538)	0.000
Node stage																
N0		1				1				1				1		
N1	−1.666	0.466	0.189(0.076–0.471)	0.000	−1.554	0.667	0.211(0.057–0.782)	0.020	−1.443	0.342	0.236(0.121–0.462)	0.000	−1.568	0.406	0.209(0.094–0.463)	0.000
N2	−1.960	0.325	0.141(0.074–0.267)	0.000	−0.742	0.387	0.476(0.223–1.017)	0.055	−1.216	0.225	0.296(0.191–0.460)	0.000	−1.392	0.258	0.249(0.150–0.412)	0.000
N3	−1.117	0.278	0.327(0.190–0.564)	0.000	−0.472	0.380	0.624(0.296–1.315)	0.215	−0.832	0.216	0.435(0.285–0.665)	0.000	−1.080	0.249	0.339(0.209–0.553)	0.000
Clinical stage																
III		1				1				1				1		
IV	−0.606	0.222	0.546(0.353–0.844)	0.006	−0.405	0.244	0.667(0.413–1.076)	0.097	−0.674	0.151	0.510(0.379–0.685)	0.000	−1.166	0.191	0.312(0.214–0.453)	0.000
Target therapy																
Without		1				1				1				1		
With	0.796	0.325	2.217(1.174–4.188)	0.014	−0.367	0.260	0.693(0.416–1.154)	0.158	0.292	0.187	1.340(0.928–1.933)	0.118	0.707	0.263	2.028(1.213–3.393)	0.007
Induction chemotherapy																
No		1				1				1				1		
Yes	0.107	0.230	1.113(0.709–1.747)	0.642	−0.354	0.270	0.702(0.413–1.193)	0.191	−0.181	0.163	0.834(0.607–1.148)	0.266	−0.279	0.204	0.756(0.507–1.128)	0.170

**Table 5:** Multivariate analysis of variables correlated with the treatment regimen status and other significant prognostic factors in 730 eligible cases

	DMFS			LRFS			PFS			OS		
	B	HR(95%CI)	*P*	B	HR (95%CI)	*P*	B	HR (95%CI)	*P*	B	HR (95%CI)	*P*
Gender												
Female	1			1			1			1		
Male	−0.934	0.393 (0.188–0.823)	0.013	0.247	1.280 (0.733–2.237)	0.385	−0.205	0.815 (0.549–1.210)	0.310	−0.523	0.592 (0.347–1.011)	0.055
Age												
<44	1			1			1			1		
≥ 44	−0.096	0.909 (0.582–1.421)	0.675	−0.439	0.645 (0.393–1.058)	0.082	−0.256	0.774 (0.571–1.050)	0.100	−0.252	0.777 (0.534–1.132)	0.189
Tumor stage												
T1	1			1			1			1		
T2	0.149	1.161 (0.335–4.026)	0.814	−0.229	0.795 (0.113–5.603)	0.818	−0.200	0.819 (0.287–2.339)	0.709	0.206	1.229 (0.407–3.714)	0.715
T3	−0.894	0.409 (0.128–1.305)	0.131	0.109	1.116 (0.246–5.065)	0.887	−0.510	0.600 (0.256–1.405)	0.240	−0.546	0.580 (0.215–1.561)	0.280
T4	−0.474	0.623 (0.248–1.562)	0.313	−0.329	0.720(0.175–2.954)	0.648	−0.664	0.515 (0.243–1.091)	0.083	−0.632	0.531 (0.231–1.223)	0.137
Node stage												
N0	1			1			1			1		
N1	−1.979	0.138 (0.046–0.418)	< 0.001	−1.437	0.238 (0.047–1.210)	0.083	−1.603	0.201 (0.088–0.458)	< 0.001	−1.509	0.221 (0.086–0.567)	0.002
N2	−2.251	0.105 (0.043–0.260)	< 0.001	−0.594	0.552 (0.160–1.902)	0.347	−1.329	0.265 (0.138–0.508)	< 0.001	−1.242	0.289 (0.141–0.594)	0.001
N3	−1.330	0.264 (0.111–0.629)	0.003	−0.329	0.720(0.204–2.542)	0.609	−0.876	0.416 (0.216–0.804)	0.009	−0.800	0.449 (0.216–0.934)	0.032
Clinical stage												
III	1			1			1			1		
IV	0.224	1.251 (0.436–3.593)	0.677	−0.052	0.950 (0.212–4.254)	0.946	0.080	1.083 (0.482–2.434)	0.847	−0.448	0.639 (0.256–1.598)	0.338
Target therapy												
Without	1			1			1			1		
With	0.840	2.317 (1.224–4.384)	0.010	−0.339	0.712 (0.427–1.189)	0.194	0.353	1.423 (0.985–2.058)	0.060	0.754	2.125 (1.269–3.560)	0.004
Induction chemotherapy												
No	1			1			1			1		
Yes	0.303	1.354 (0.849–2.159)	0.203	−0.209	0.811 (0.471–1.398)	0.451	0.031	1.031 (0.742–1.434)	0.855	−0.012	0.988 (0.655–1.490)	0.955

## Discussion

Even with the administration of cisplatin-based chemoradiotherapy, the treatment outcome for advanced stages of NPC is still unsatisfactory because of local recurrence and/or distant metastasis, which represent the major patterns of disease failure [17]. However, modern radiation techniques and equipment have enabled the delivery of high doses of radiation to the target tissue while sparing normal organs from risk, thereby potentially enhancing the therapeutic efficacy [18]. Previous studies have reported 90% local-regional control rates for nasopharyngeal carcinoma with the use of IMRT combined with systematic chemotherapy, even in patients presenting with advanced loco-regional disease [19–21]. Distant metastasis plays an important role in treatment failure and needs to be managed properly and urgently. After decades of studies on chemotherapy for NPC, only slight improvements have been achieved in survival and distant failure; therefore, new treatment strategies must be developed to address this issue, which has confounded clinical doctors for a long time.

With further research of the molecular mechanism of tumorigenesis and tumor development, molecular targeted therapy in patients with NPC has become a research hotspot. It is known that more than 90% patients with NPC were positive for the overexpression of EGFR [6, 7], which is considered an important target in NPC treatment [22]. Nimotuzumab is a humanized anti-EGFR monoclonal antibody, and it is obtained by replacing a murine complementary-determining region with a human framework. Nimotuzumab has shown high safety and low toxicity without the severe skin and mucosa toxicities commonly associated with other EGFR-targeting antibodies [12, 15]. As reported, compared with other EGFR inhibitors, such as cetuximab, nimotuzumab shows a greater advantage in terms of less toxicity [23]. Another advantage of nimotuzumab is that the affinity constant is quite low, which allows for high tumor uptake and low normal tissue uptake. Research has shown that Nimotuzumab demonstrates marked antiproliferative, proapoptotic, and antiangiogenic effects in tumors that overexpress EGFR [24]. Currently, Nimotuzumab has been approved in several countries for the treatment of head and neck tumors [25, 26].

The current study retrospectively analyzed the efficacy of nimotuzumab plus IMRT/CCRT with or without induction chemotherapy in 184 NPC patients. In our study, encouraging survival rates and distant metastasis control were attributed to the treatment with nimotuzumab. Our results showed promising clinical outcomes, with a 5-year DMFS of 93.09%, 5-year LRRFS of 85.34%, 5-year PFS of 79.96%, and 5-year OS of 88.91% observed in patients who received nimotuzumab and a 5-year DMFS of 85.61%, 5-year LRRFS of 89.79%, 5-year PFS of 77.99%, and 5-year OS of 78.30% in patients who did not receive nimotuzumab. The lack of significant difference in the 5-year LRRFS (85.34% vs. 89.79%, *P* = 0.156) was reasonable since IMRT provides excellent locoregional control [27]. The current analysis demonstrated that the addition of nimotuzumab compared with CCRT alone was associated with a significantly better OS and DMFS, which presented significantly differences of *P* = 0.006 and *P* = 0.012, respectively. Further statistical analyses showed that OS significantly increases in patients with stage III disease. These data indicated that the increase in survival outcome for NPC patients treated with nimotuzumab was mainly attributed to the significant increase in DMFS, which could be related to the greater ability of nimotuzumab and cisplatin-based chemoradiotherapy to kill tumor cells, especially cisplatin-based chemotherapy-resistant micrometastases. This improved tumor killing ability could also partially explain the significant increase in DMFS; however, this is just a postulation, and further investigation is required to explore the exact mechanism.

Previous studies demonstrated that the main prognostic factors for survival are age, gender, T and N category, and clinical stage, with the survival rate decreasing as the T category and N category increased [28]. According to the multivariate analysis, gender, N stage and nimotuzumab were significant prognostic factors for DMFS; N stage and nimotuzumab treatment were significant prognostic factors for OS; and node stage was a significant prognostic factor for PFS. Since only patients with clinical stage III and IV were included in this study and the local-regional control rate was similar and lacked statistical significance (90.16% vs. 85.71%, *P* = 0.156), these results can be explained by the use of modern radiation techniques, which have been proved to improve local-regional control. For distant failure, node stage still affects DMFS and OS, and patients with advanced node stage have a higher likelihood of distant failure, which leads to overall failure. These results are consistent with that of other studies [5, 29]. We must address the significant improvement of overall survival after the administration of a full course of nimotuzumab to NPC patients in stages III to IV during chemoradiotherapy, with these patients showing a nearly 10% improvement in OS (88.91% vs 78.30%, *P* = 0.006). The results are encouraging and beyond our expectations. The strength of nimotuzumab combined with radiotherapy for NPC may be still largely due to the strengthening of the antitumor effect caused by the increased tumor cell killing ability of nimotuzumab and cisplatin-based chemoradiotherapy, which was mentioned above.

This study presented certain limitations, and the results should be interpreted with caution since this is a retrospective study. Moreover, the lack of availability of EGFR expression data is another limitation since a proportion of patients were EGFR negative. Although we eliminated selection bias, such as gender, age, T and N stage, and clinical stage, using propensity scores, whether other confounding factors still exist remains unclear. In the future, prospective, randomized, well-designed, and large-sample clinical studies are needed to evaluate these factors.

## Conclusions

In conclusion, our study observed that the administration of nimotuzumab during chemoradiotherapy in stage III-IV NPC patients showed promising clinical outcomes compared with the administration of chemoradiotherapy alone. However, additional studies, especially prospective, well-designed, and large-sample clinical studies, are needed.

## Data Availability

The data of this research (RDDA2019001088) is deposited in RDD (http://www.researchdata.org.cn).

## Abbreviations

AJCC: American Joint Committee on Cancer
CCRT: Concurrent chemoradiotherapy
CT: Computed tomography
CTV: Clinical tumor volume
DMFS: Distant metastasis–free survival
EGFR: Epidermal growth factor receptor
GTVnx: The primary nasopharyngeal gross tumor volume
IC: Induction chemotherapy
IMRT: Intensity-modulated radiotherapy
KPS: Karnofsky Performance Status
LRRFS: Locoregional relapse–free survival
MRI: Magnetic Resonance Imaging
NPC: Nasopharyngeal carcinoma
OS: Overall survival
PET-CT: Positron emission tomography–computed tomography
PFS: Progression–free survival
PTV: Planning target volume
RTOG: Radiation Therapy Oncology Group
SYSUCC: Sun Yat-sen University Cancer Center
